# The impact of a novel deep learning reconstruction algorithm on image quality in ultralow-dose CT: a quantitative phantom study

**DOI:** 10.1186/s41747-026-00751-w

**Published:** 2026-06-08

**Authors:** Tong Su, Yongjun Jia, Yun Shen, Huairong Zhang

**Affiliations:** 1 Computed Tomography Business Unit, Neusoft Medical Systems Co., Ltd., Shenyang, China; 2 Department of Radiology, Affiliated Hospital of Shaanxi University of Chinese Medicine, Xianyang City, China; 3 Department of Radiology, General Hospital of Ningxia Medical University, Yinchuan, China

**Keywords:** Algorithms, Contrast media, Deep learning, Phantoms (imaging), Radiation dosage, Tomography

## Abstract

**Objective:**

The aim of this study is to evaluate the performance of a novel deep learning image reconstruction (DLIR) algorithm in noise reduction, contrast-to-noise ratio (CNR), and low iodine concentration detection for ultralow-dose computed tomography (CT) imaging.

**Methods:**

A nine-hole phantom with iodine concentrations (0–40 mg/mL) was scanned at various tube voltages (60–120 kVp). Images were reconstructed using filtered back projection (FBP), iterative reconstruction (IR), and DLIR at different weight levels (10%–90%). Objective metrics (noise, CNR, CT value accuracy via Bland-Altman analysis) and subjective image quality were assessed.

**Results:**

At all tube voltages (60–120 kVp), DLIR with medium-to-high weight levels (50%–90%) reduced background noise and increased CNR compared with FBP and IR (*p* < 0.001). The CNR at a low iodine concentration (1.25 mg/mL) was enhanced, and the DLIR algorithm (weight levels 30%-90%) was able to continuously detect an iodine concentration of 1.25 mg/mL (CNR ≥ 3) at all tube voltages. Under fixed ultralow-dose conditions, DLIR preserved image quality and low-contrast detectability. DLIR (weight levels 90%) reduced background noise by 84.7% compared with FBP and improved CNR (*p* < 0.001). Bland-Altman analysis confirmed excellent quantitative accuracy for DLIR. The exploratory subjective evaluation was consistent with objective metrics.

**Conclusion:**

The DLIR algorithm can enhance image quality in low-dose CT imaging and improve the ability to detect low concentrations of iodine. These findings demonstrate that DLIR maintains image quality and CNR at low iodine concentrations in phantom studies. Clinical implications require further validation.

**Relevance statement:**

This phantom study shows that the deep learning reconstruction algorithm can still maintain the diagnostic image quality and low-contrast detectability even under ultralow-dose CT (94% dose reduction). These findings support further clinical research to optimize the dosage regimens and potentially reduce the use of iodine contrast agents.

**Key Points:**

- Under ultralow-dose conditions (60 kV), DLIR preserved image quality metrics and detectability thresholds in a phantom under ultralow-dose conditions.
- It significantly suppressed image noise and improved the CNR.
- The algorithm reliably detected low iodine concentrations (1.25 mg/mL) at all dose levels.

**Graphical Abstract:**

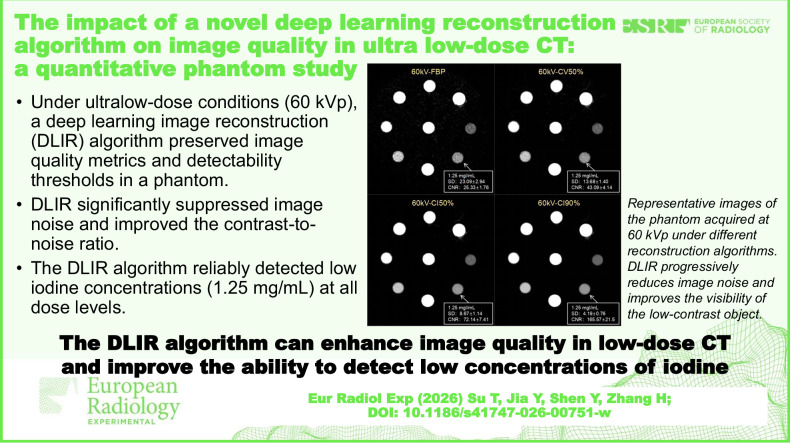

## Background

With the widespread use of computed tomography (CT) in disease screening and follow-up, the risk of accumulated radiation dosage has increasingly become a focal point of clinical concern, which drives the clinical imperative for low-dose CT protocols to reduce radiation risk for patients [1, 2]. However, the reduction in dose often leads to increased image noise and reduced contrast, which can affect image quality. Especially in cases of low iodine concentration, it is prone to miss small lesions (such as early pulmonary nodules or low-enhancement lesions) [3–5]. Traditional filtered back projection (FBP) and iterative reconstruction (IR) algorithms have certain limitations under ultralow-dose conditions, such as blurred details and unclear texture display, which restrict their clinical application [6–9].

In recent years, deep learning technology has made significant breakthroughs in the field of medical imaging. The image reconstruction algorithm based on convolutional neural networks provides a new solution for optimizing the radiation dose in CT imaging. The deep learning image reconstruction (DLIR) technology enables convolutional neural networks to learn the mapping relationship between noise and the true anatomy. By leveraging the feature extraction and learning capabilities of neural networks, it significantly reduces noise while preserving anatomical details [10–13]. For instance, the research conducted by Jo et al [11] demonstrated that, compared with the conventional dose IR, even when the dose was reduced by 75%, DLIR was still able to maintain image quality and the detectability of nodules. However, there are still significant deficiencies and gaps in the current systematic evaluation of DLIR under ultralow-dose conditions, particularly in research regarding the critical clinical application scenario of low-contrast iodine detection. Iodine contrast agents play a crucial role in vascular imaging, tumor detection, and CT assessment of organ function. Low-dose scans often lead to an increase in image noise, making it difficult to detect lesions with low iodine concentrations [14–16].

This study used a nine-hole phantom to evaluate the application of a clinically feasible DLIR algorithm in ultralow-dose CT, with a particular focus on its capabilities in noise suppression, contrast enhancement, and low iodine concentration detection. We quantitatively assessed its performance in noise reduction, contrast-to-noise ratio (CNR) improvement, and low-iodine-concentration detectability across varying tube voltages (60–120 kVp) and iodine concentrations (1.25–10 mg/mL), comparing it with FBP and IR algorithms. Our aim is to determine whether this DLIR algorithm can maintain diagnostic image quality under substantially reduced dose conditions, especially showing improvements in detecting lesions with low iodine concentration.

## Methods

### Phantom parameters

**Fig. 1:**
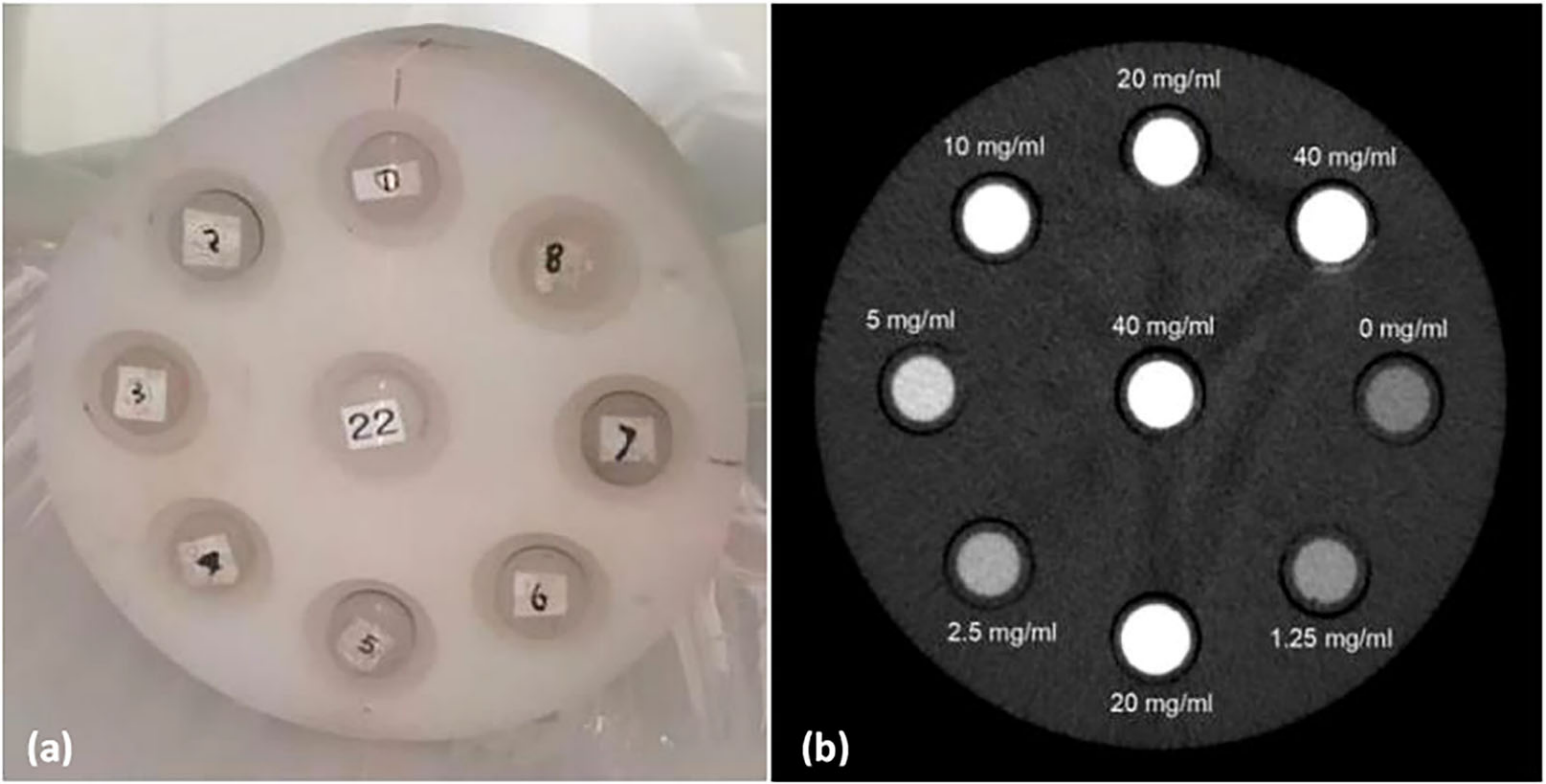
**a** The QSP Phantom contains nine test tubes. **b** The test tubes are filled with iodinated contrast agent solutions of different concentrations: 0 (water), 1.25, 2.5, 5, 10, 20 (for three test tubes), and 40 mg/mL

We employed a custom cylindrical QSP model (Designed and fabricated in-house, Tokyo, Japan) (Fig. 1), which is composed of an epoxy resin base with a diameter of 200 mm. Inside the base, nine hard plastic tubes with a diameter of 20 mm and a length of 100 mm are arranged in a circular pattern. These tubes are filled with iodinated contrast agent solutions of different concentrations: 0 (water), 1.25, 2.5, 5, 10, 20 (for three test tubes), and 40 mg/mL. This is to simulate the unenhanced background and different degrees of contrast enhancement. This design enables systematic evaluation of low-contrast detectability and image quality preservation under low-dose conditions. The modular design also allows for future extensions to evaluate other parameters.

### Image acquisition and reconstruction

The QSP phantom was scanned using the NeuViz Epoch CT (16 cm wide-body detector, 256 rows, Neusoft Medical Systems Co., Ltd). Tube voltages and the corresponding radiation dose levels are fixed experimental settings, and no dose optimization or equivalence analysis was performed. Tube voltages were set to four levels: 120, 100, 80, and 60 kVp, with a reference tube current of 100 mAs. The actual tube current is automatically adjusted by the O-Dose platform. The slice thickness was 1 mm, the rotation time was 0.4 s, and the pitch was 0.8. The corresponding CT dose index volume (CTDI_vol_) values were 0.33 mGy (60 kVp), 1.38 mGy (80 kVp), 3.26 mGy (100 kVp), and 5.73 mGy (120 kVp). Compared to the 120 kVp reference protocol, the CTDI_vol_ values for the 100, 80, and 60 kVp protocols were reduced by 43.1%, 75.9%, and 94.2%, respectively. For the 120, 100, and 80 kVp groups, reconstructed images were processed using FBP, iterative algorithms, and DLIR. The IR was configured with 50% weighting for ClearView (CV), while the DLIR reconstruction utilized ClearInfinity (CI) algorithms with 10%, 30%, 50%, 70%, and 90% weighting settings. For the 60 kVp group, FBP, CV, and CI images were reconstructed. The weights for CV and CI were set at 10%, 30%, 50%, 70%, and 90%, respectively.

### Principle of the deep-learning image reconstruction algorithm

CI is a commercially available DLIR algorithm (Neusoft Medical Systems) that has received the U.S. Food and Drug Administration (FDA) and the National Medical Products Administration of China (NMPA) clearance. It provides 10 weight levels (0% to 90% in 10% increments) for image reconstruction, allowing noise reduction and image quality to be adjusted based on diagnostic needs and dose limits. This proprietary algorithm uses a deep convolutional neural network trained on paired high- and low-quality CT data collected from partner hospitals under ethical supervision. It effectively separates structural information from noise and artifacts to enhance image reconstruction. As the algorithm is proprietary, specific details of the training dataset (*e.g*., size, source distribution) cannot be independently verified.

### Image quality evaluation

#### Objective evaluation

In the reconstructed image groups, a region of interest of the same size (100 mm²) was selected at the center of the phantom tube to cover 80% of the phantom aperture and avoid edge effects. Since only one acquisition was performed per scan parameter combination, to mitigate the impact of random variation, the mean and standard deviation (SD) of the CT values were measured over 15 consecutive slices for each tube, and the average was calculated. However, this method still cannot fully reflect the stability of the equipment noise or the repeatability of the experiment. Noise is defined as the average value of the 15-slice SD values. With 0 mg/mL as the background, select test tubes with concentrations of 1.25, 5, and 10 mg/mL as the targets, and calculate the CNR for each iodine concentration test tube.

$${{\rm{CNR}}}=\frac{\left|{{{\rm{CT}}}}_{{{\rm{Iodine}}}}-{{{\rm{CT}}}}_{{{\rm{background}}}}\right|}{{{{\rm{SD}}}}_{{{\rm{background}}}}}$$

We set a CNR ≥ 3 as the threshold standard for iodine concentration detectability, and recorded the minimum reconstruction condition under which 1.25 mg/mL could be identified [17]. Radiation dose: Record the CTDI_vol_ and the dose-length product for each scan.

#### Subjective evaluation

Two radiologists (each with > 5 years of experience) evaluated the images in a double-blind manner under standardized display settings. The overall image quality was evaluated using a 5-point Likert scale (see Supplementary Table S1 for detailed criteria), with a focus on assessing the presence of image noise and artifacts. This subjective assessment is merely an exploratory supplementary evaluation and does not serve as an independent criterion for evaluating image quality.

### Statistical analysis

Statistical analysis was conducted using SPSS 26.0 (IBM Corp.). The differences between groups were analyzed using one-way analysis of variance or Kruskal-Wallis H test; The Bland-Altman analysis was used to evaluate the consistency of the quantitative accuracy of CT values from deep learning reconstructed images; Spearman correlation analysis was employed to investigate the relationships between radiation dose, reconstruction algorithms, and image quality indicators. The threshold effect was analyzed using a heat map, which visually summarized the optimal parameter combinations that met the iodine detection standard (CNR ≥ 3). No formal statistical inferences could be drawn from this. Two radiologists evaluated the images subjectively using the intraclass correlation coefficient to assess consistency. The inter-group scores were analyzed using non-parametric tests, and pairwise comparisons were performed with Bonferroni correction. A *p* value < 0.050 was considered statistically significant.

## Results

### Objective image quality assessment

Among all the tube voltage groups, as the tube voltage decreased, the background noise significantly increased. The SD value of the 120 kVp-FBP group was 12.94 ± 0.95 HU, while that of the 60 kVp-FBP group rose to 23.74 ± 1.57 HU (*p* < 0.001). For all voltage groups, both the iterative algorithm and the DLIR algorithm significantly reduce background noise and image noise (SD value) in the test tubes with varying iodine concentrations. The effect is better than FBP (*p* < 0.001), and the denoising effect increases with the weight of the CI algorithm (*p* < 0.001).

**Fig. 2:**
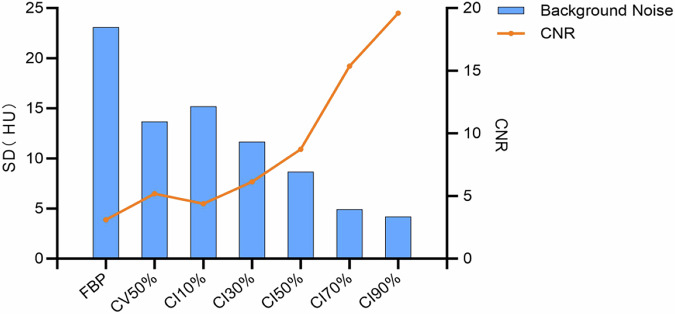
Image noise and CNR across different reconstruction algorithms at 60 kVp. The bar graph (left *y*-axis) shows the background noise (SD value), while the line graph (right *y*-axis) shows the CNR for the 1.25 mg/mL iodine insert. As the weight level of the DLIR algorithm increases (CI 50%–90%), image noise is significantly reduced, and the CNR is significantly enhanced. CI, ClearInfinity; CNR, Contrast-to-noise ratio; CV, ClearView; DLIR, Deep learning image reconstruction; FBP, Filtered back projection

In all test tubes with iodine concentrations (1.25, 5, 10 mg/mL), the CNR value significantly increased with the increasing CI algorithm weight (*p* < 0.001, Table 1, Fig. 2). At the fixed ultra‑low‑dose setting (60 kVp), the DLIR algorithm reduced noise and increased CNR compared with FBP. For a low iodine concentration of 1.25 mg/mL, under the conventional dose (120 kVp), the CNR of the CI 90% reconstruction (11.12 ± 1.28) reached 4.5 times that of the FBP reconstruction (2.49 ± 0.17).

**Table 1:** Comparison of CNR and iodine detection thresholds under different reconstruction algorithms for different tube voltage groups

	FBP	CV 50%	CI 10%	CI 30%	CI 50%	CI 70%	CI 90%	*p*-values
CNR (1.25 mg/mL)								
120 kVp	2.49 ± 0.17	3.52 ± 0.27	2.83 ± 0.22	3.81 ± 0.30	5.48 ± 0.41	9.36 ± 0.82	11.12 ± 1.28	< 0.001
100 kVp	2.62 ± 0.21	3.86 ± 0.27	3.21 ± 0.21	4.20 ± 0.35	5.99 ± 0.39	9.91 ± 0.79	12.55 ± 1.72	< 0.001
80 kVp	2.36 ± 0.27	3.23 ± 0.33	2.80 ± 0.26	3.62 ± 0.33	5.16 ± 0.45	8.72 ± 0.76	10.55 ± 1.42	< 0.001
60 kVp	3.11 ± 0.29	5.19 ± 0.6	4.38 ± 0.87	6.13 ± 1.57	8.72 ± 0.96	15.36 ± 2.06	19.59 ± 2.64	< 0.001
CNR (5 mg/mL)								
120 kVp	10.48 ± 0.73	14.92 ± 1.15	11.84 ± 0.96	15.99 ± 1.25	23.00 ± 1.61	39.67 ± 3.81	48.02 ± 5.68	< 0.001
100 kVp	11.14 ± 0.81	16.39 ± 1.17	13.46 ± 0.69	17.89 ± 1.08	25.14 ± 1.47	41.65 ± 3.01	54.35 ± 7.24	< 0.001
80 kVp	12.28 ± 0.83	17.32 ± 1.38	14.70 ± 1.09	18.98 ± 1.25	26.93 ± 1.85	45.56 ± 3.75	56.58 ± 7.09	< 0.001
60 kVp	12.81 ± 0.93	21.68 ± 2.09	19.17 ± 1.44	25.98 ± 2.92	36.36 ± 3.86	64.30 ± 8.30	83.33 ± 10.9	< 0.001
CNR (10 mg/mL)								
120 kVp	20.35 ± 1.46	28.99 ± 2.22	22.96 ± 1.89	31.03 ± 2.39	44.63 ± 3.11	76.31 ± 6.30	93.37 ± 10.9	< 0.001
100 kVp	21.74 ± 1.58	32.17 ± 2.21	26.34 ± 1.38	34.97 ± 2.15	48.21 ± 4.91	81.39 ± 6.07	106.72 ± 14.3	< 0.001
80 kVp	24.62 ± 1.62	34.86 ± 2.74	29.49 ± 2.17	38.1 ± 2.56	53.98 ± 3.74	91.38 ± 7.59	114.33 ± 14.4	< 0.001
60 kVp	25.33 ± 1.76	43.09 ± 4.14	37.74 ± 2.83	51.45 ± 5.69	72.14 ± 7.41	127.07 ± 15.9	165.57 ± 21.5	< 0.001
Iodine detection thresholds (mg/mL)								
120 kVp	5	1.25	5	1.25	1.25	1.25	1.25	
100 kVp	5	1.25	1.25	1.25	1.25	1.25	1.25	
80 kVp	5	1.25	5	1.25	1.25	1.25	1.25	
60 kVp	1.25	1.25	1.25	1.25	1.25	1.25	1.25	

CNR data are given as mean ± SD. The *p*-values for each row (under specific tube voltage and iodine concentration conditions) indicate the significant test results of the overall differences in CNR among the 7 different reconstruction algorithms. The iodine detection threshold refers to the lowest iodine concentration (mg/mL) corresponding to the minimum CNR value that meets the detectable standard (CNR ≥ 3) under the conditions of this row. The lowest concentration tested in this study was 1.25 mg/mL

*CI* ClearInfinity, *CNR* Contrast-to-noise ratio, *CV* ClearView, *FBP* Filtered back projection

**Fig. 3:**
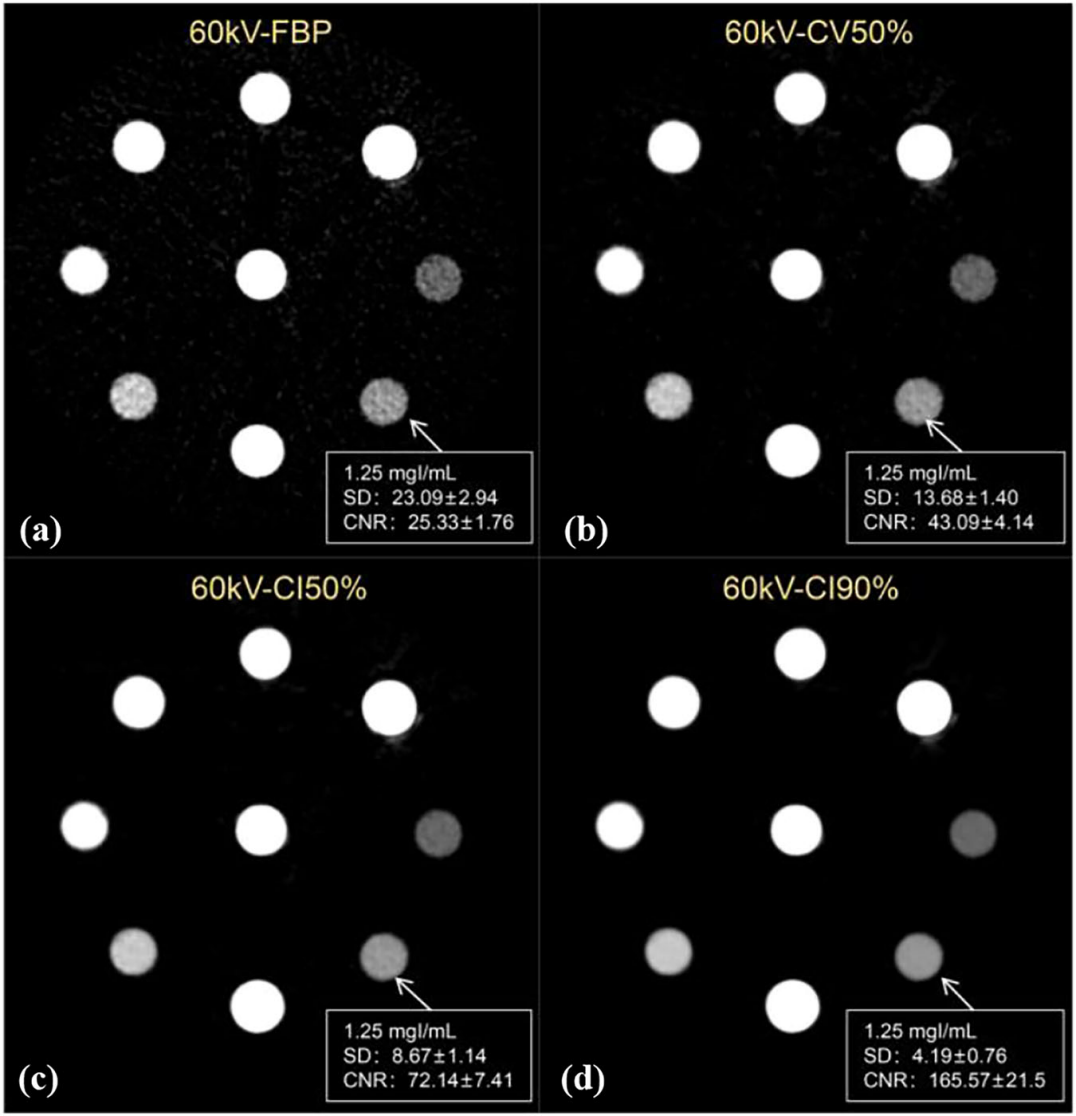
Representative images of the phantom acquired at 60 kVp under different reconstruction algorithms; (**a**) FBP, (**b**) CV50%, (**c**) CI50%, and (**d**) CI90%. All images are displayed at the identical window width and level (*e.g*., window width 400, window level 40). The arrow points to the 1.25 mg/mL insert. The DLIR algorithm progressively reduces image noise and improves the visibility of the low-contrast object. CI, ClearInfinity; CNR, Contrast-to-noise ratio; CV, ClearView; DLIR, Deep learning image reconstruction; FBP, Filtered back projection

Based on the iodine detection standard of CNR ≥ 3, at a conventional dose (120 kVp), the FBP algorithm and the low-weight DLIR algorithm (CI 10%) have an iodine detection lower limit of only 5 mg/mL, making it impossible to consistently detect low-concentration iodine solutions at 1.25 mg/mL. However, with the medium-to-high-weight DLIR algorithm (CI 50%–90%), the iodine detection threshold of 1.25 mg/mL was consistently achieved across the entire dose range from 120 to 60 kVp. At ultralow dose (60 kVp), all algorithms (including FBP) can detect 1.25 mg/mL, but the FBP algorithm exhibits high image noise (SD: 23.74 ± 1.57 HU) and low CNR (3.11 ± 0.29); In contrast, the DLIR algorithm (using CI 90% as an example) exhibited low noise (SD: 3.63 ± 0.48 HU) and substantially increased CNR (19.59 ± 2.64) (Tables 1 and 2 and Fig. 3).

**Table 2:** Bland-Altman agreement analysis comparing various CI reconstruction levels against the reference standard (60 kVp-CV 50%) at the ultralow-dose 60 kVp setting

Reconstruction agreement	1.25 mg/mL		5 mg/mL		10 mg/mL	
	Bias (HU)	95% LoA (HU)	Bias (HU)	95% LoA (HU)	Bias (HU)	95% LoA (HU)
60 kVp-CI 10%	0.40	-3.44, +4.24	-3.15	-8.47, +2.17	-1.43	-4.56, +1.69
60 kVp-CI 30%	-0.73	-4.15, +3.33	-1.49	-6.15, +3.17	-1.43	-3.77, +0.90
60 kVp-CI 50%	0.42	-2.48, +3.33	-1.96	-6.63, +2.70	0.20	-1.26, +1.65
60 kVp-CI 70%	0.10	-2.78, +2.98	-1.98	-6.25, +2.29	-1.31	-4.59, +1.97
60 kVp-CI 90%	2.24	-0.41, +4.89	1.76	-3.38, +6.89	2.48	-0.86, +5.83

The table presents the mean bias (in HU) and the 95% limits of agreement (LoA) for values measurements across three iodine concentrations (1.25, 5, and 10 mg/mL). Each row represents a comparison between different reconstruction levels (from CI 10% to CI 90%) and the iterative reference standard (CV 50%). Bias values close to zero and narrow LoA indicate closer agreement with the reference

*Bias Mean bia* mean difference between the test and reference methods, *CI* ClearInfinity, *CV* ClearView

### Bland-Altman evaluation of quantitative accuracy of CT values

**Fig. 4:**
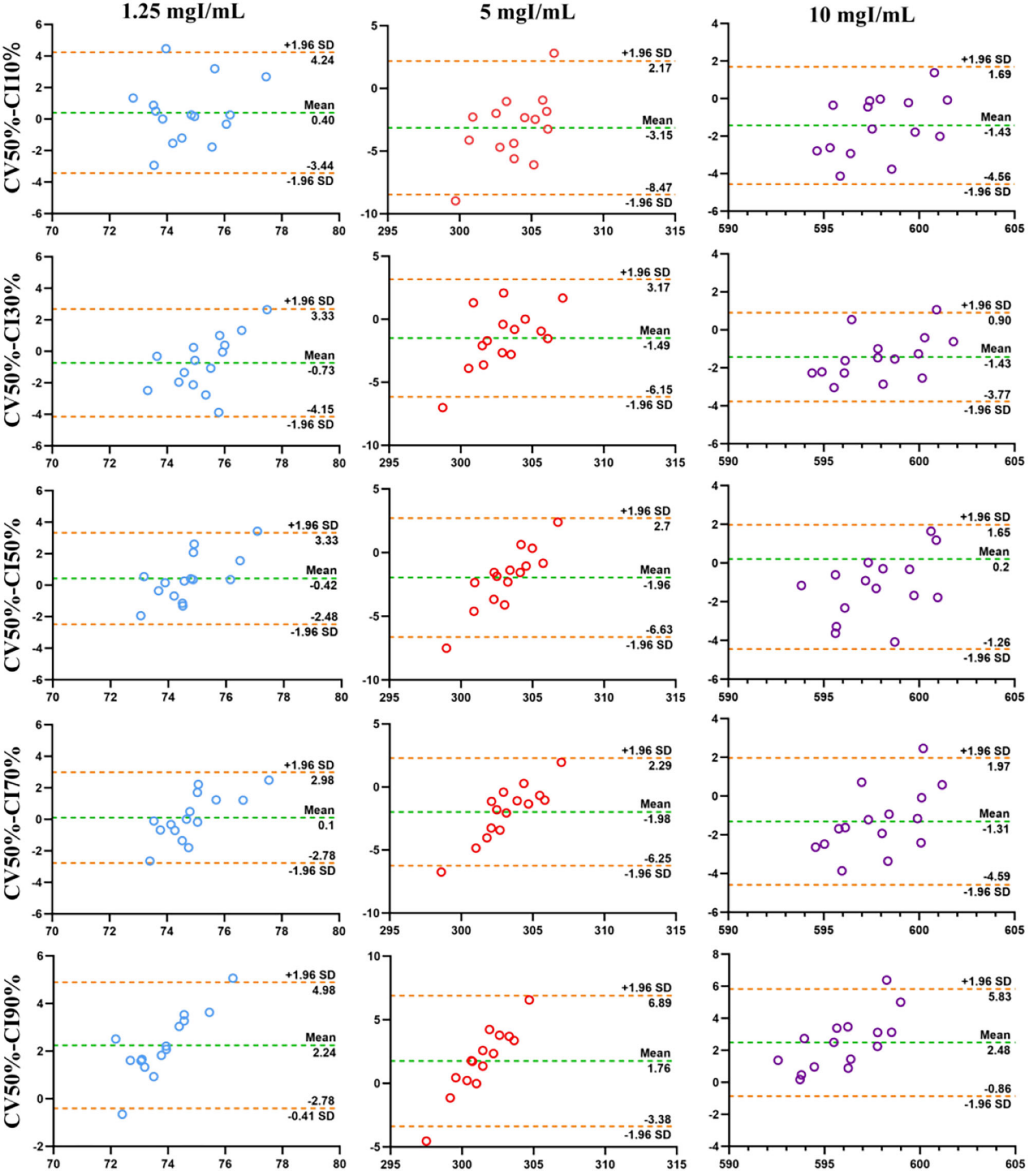
Bland-Altman plots for three iodine concentrations (1.25, 5, and 10 mgI/mL) at 60 kVp under different DLIR reconstruction algorithms (from CI 10% to CI 90%), using 60 kVp-CV50% images as the reference standard. CI, ClearInfinity; DLIR, Deep learning image reconstruction

To accurately assess the impact of the DLIR algorithm on the quantitative accuracy of CT values, we employed Bland-Altman analysis. The results are shown in Table 2 and Fig. 4, where the absolute values of the mean bias of the low and medium-weight DLIR algorithms (CI 10%–CI70%) compared to the reference standard are all less than 3.2 HU at different iodine concentrations, and the overall 95% limits of agreement (LoA) are relatively narrow. Taking the low concentration of 1.25 mg/mL as an example, the mean bias for CI 70% is only 0.10 HU, with the 95% LoA ranging from -2.78 to +2.98 HU. The highest weight algorithm CI 90% exhibits a trend distinct from other intensities, consistently showing a positive bias (ranging from 1.76 to 2.48 HU) across three iodine concentrations. However, the bias values are relatively small, and the 95% LoA remains within clinically acceptable limits in all cases (the widest being -3.38 to +6.89 HU).

### Results of relevance analysis and threshold effect analysis

To deeply explore the intrinsic relationships among various variables, we conducted a Spearman rank correlation analysis. Among all reconstruction algorithms, the CTDI_vol_ is significantly negatively correlated with background noise (SD value) (all *p* < 0.001). As the weight levels of the DLIR algorithm increase, the intensity of this negative correlation gradually weakens (Spearman’s ρ decreases from -0.928 for FBP to -0.629 for CI90%). Under any fixed tube voltage conditions, different DLIR reconstruction algorithms show a strong and significant positive correlation with the CNR value of 1.25 mg/mL (120 kVp: ρ = 0.924; 100 kVp: ρ = 0.937; 80 kVp: ρ = 0.930; 60 kVp: ρ = 0.937; all *p* < 0.001).

**Fig. 5:**
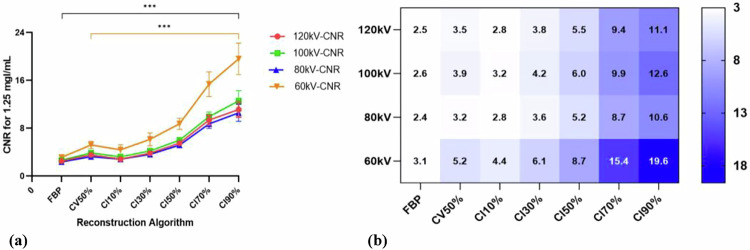
**a** The trend graph of CNR (1.25 mg/mL) varying with the weight levels of the DLIR reconstruction algorithm under different power supply voltages. **b** Heatmap of the detection capability for 1.25 mg/mL iodine based on a CNR ≥ 3 across different tube voltages and reconstruction algorithms. CI, ClearInfinity; CNR, Contrast-to-noise ratio; CV, ClearView; FBP, Filtered back projection

As shown in Fig. 5a, the line graph clearly shows that as the algorithm weight increases, the CNR value significantly rises. Especially under ultralow dose (60 kVp) conditions, the improvement is the most obvious. Even with the lowest weight DLIR algorithm (CI 10%) at 60 kVp, its CNR value has surpassed that of the traditional dose FBP reconstruction (120 kVp-FBP). The Wilcoxon rank-sum test indicated that the CNR in the 60 kVp-CI 90% group was significantly higher than that in the 120 kVp-FBP group, the 60 kVp-FBP group, and the 60 kVp-CV 50% group (*p* < 0.001).

Based on the threshold of CNR ≥ 3, we plotted a heatmap of the detection capability for an iodine concentration of 1.25 mg/mL under different tube voltages and reconstruction parameters, as shown in Fig. 5b. The results show that under ultralow dose conditions of 60 kVp, all reconstruction algorithms (including the traditional FBP) can achieve detection at 1.25 mg/mL, primarily due to the significant enhancement in iodine contrast brought about by low tube voltage scanning. Under conventional and medium-low dose conditions (120, 100, or 80 kVp), the traditional FBP algorithm fails to reach the detection threshold, while CI 30% is the minimum algorithm strength required for stable detection. However, the medium to high weight deep learning reconstruction algorithms (CI 50%–90%) successfully expanded the detection limit to 1.25 mg/mL.

### Subjective image quality evaluation

This subjective evaluation serves as an exploratory and supplementary study, and the results obtained shall not be used to assess its clinical performance. The consistency between the two raters is excellent, with an intraclass correlation coefficient of 0.853 (95% confidence interval: 0.80–0.89). There were significant differences in the overall subjective scores among the images of different reconstruction algorithm groups (*p* < 0.001), with CI 50%–90% receiving higher scores than FBP and CV 50% across all dose levels (all *p* < 0.050, Bonferroni-corrected). Detailed score distributions and pairwise comparisons are provided in Supplementary Table S2.

## Discussion

This phantom study demonstrates that the DLIR algorithm can effectively maintain diagnostic image quality under ultralow-dose CT conditions. Our quantitative research results indicate that for DLIR (particularly at medium to high weight levels, CI 50%–90%), within the tube voltage range of 60–120 kVp, it can reduce image noise and enhance the CNR compared to traditional FBP and IR. A key finding is that DLIR was able to continuously detect low iodine concentrations (1.25 mg/mL) at all dose levels (including ultralow dose conditions, which corresponded to a 94.2% reduction from the conventional dose regimen).

Previous studies have shown that the DLIR technology can maintain the diagnostic image quality under scanning conditions with significantly reduced radiation dose. In our study, the experimental design employed a combination of ultralow tube voltage (60 kVp) and high-weight DLIR algorithm (CI 50%–90%). Compared with the traditional 120 kVp protocol, this ultralow dose protocol reduces radiation dose by 94.2%, which is greater than the results of most previous research reports [18–27]. The DLIR technology not only successfully maintained the diagnostic image quality but also achieved a slight improvement in image quality. The body phantom study by Park et al [22] demonstrated that by using the DLIR algorithm, the radiation dose could be reduced by approximately 70% while maintaining the image quality unchanged. Compared with the FBP and IR algorithms, DLIR exhibited lower image noise, higher CNR, and superior overall image quality in both subjective and objective evaluations. Lyu et al [23] evaluated the performance of low-dose dual-energy CT with the DLIR algorithm and standard-dose single-energy CT in image quality and liver metastases detection. The results show that when the detection dose is reduced by 34%, dual-energy CT combined with DLIR technology can maintain the same image quality and liver metastases detection rate as full-dose single-energy CT. These findings are consistent with the results of our current study. Consistent with the objective results, radiologists favored CI 50–90% images over FBP or IRs, indicating DLIR can meet diagnostic needs, though patient studies are needed for further validation [22, 28, 29]. This further highlights the potential of the DLIR algorithm in optimizing the balance between radiation dose and image quality. Especially when combined with low tube voltage scanning, it can achieve “ultralow dose” and “diagnostic-level image quality”, and the images possess clinical diagnostic applicability.

Another important aspect of this study is the evaluation of iodine detection capability. The DLIR can stably detect low iodine concentrations (1.25 mg/mL) at all dose levels, whereas traditional FBP algorithms cannot attain this objective. At low iodine concentrations and low doses, this improvement is particularly beneficial. This enhancement in the detection ability for low-concentration substances suggests potential clinical relevance; however, whether this translates to improved diagnostic accuracy for tiny lesions requires dedicated clinical studies. Whether this could enable the reduction of contrast agent usage remains to be tested in clinical settings. [30–33]. This advantage stems from the unique technical mechanism of DLIR. The DLIR algorithm used in this study either has a more advanced network architecture and optimization strategies, compared to the traditional FBP and IR algorithms. It can more effectively handle the high noise in ultralow dose images and retain the effective information with diagnostic value. Literature reports indicate that conventional IR algorithms typically reduce noise by 30%–50%, while phantom-based IR algorithms primarily achieve noise reduction effects ranging from 50% to 70% [34–36]. The CI algorithm in this study offers adjustable weights ranging from 10% to 90%. Higher weight levels (*e.g*., CI 90%) maximize noise suppression but may result in slight smoothing of image texture [37]. Therefore, clinical applications should select appropriate weight levels based on diagnostic requirements: medium weight levels (CI 50%–70%) may be chosen for examinations requiring fine-structure observation to balance noise suppression and detail preservation; high weight levels (CI 80%–90%) can be employed for examinations focusing on large lesions or vascular structures to achieve optimal image quality [38–40]. In addition, the Bland-Altman analysis in this study also confirmed that DLIR can maintain high accuracy and consistency in CT value measurements while achieving noise reduction, ensuring its reliability in quantitative diagnosis and follow-up evaluations.

Therefore, the above phantom-based findings indicate that DLIR can not only preserve image quality under low-dose conditions but also exert a certain optimizing effect on various objective image quality indicators.

It is important to acknowledge the limitations of this phantom study. Firstly, as a phantom study, although the results of this research are objective and reproducible, further clinical studies are still needed to evaluate the diagnostic efficacy in clinical scenarios involving patients with complex anatomical structures and various body types. Second, the size of the object is a crucial factor in clinical imaging. Its impact on the detectability of low contrast has not been evaluated, which is an inherent limitation of the phantom design. Third, only a single acquisition was performed per condition; therefore, the random fluctuations or noise stationarity of the scanner output could not be evaluated. Future research should include repeated collection to evaluate the reproducibility of real measurements. Finally, as a phantom-based study, the visualization of fine anatomical texture structures by high-weight DLIR and its potential impact on clinical diagnostic efficacy still need to be verified through patient studies.

In conclusion, this phantom study demonstrates that the DLIR algorithm effectively reduces image noise and improves contrast‑to‑noise ratio across a range of tube voltages and low iodine concentrations. Medium‑to‑high reconstruction weights (CI50%–CI90%) consistently achieved the threshold for iodine detectability (CNR ≥ 3), while preserving quantitative CT accuracy. These findings are limited to phantom experiments and highlight the need for future clinical studies to confirm whether similar benefits can be achieved in patient imaging.

## Supplementary information

**Additional file 1:** **Table S1.** 5-point Likert scale for subjective image quality assessment. **Table S2.** Subjective evaluation scores of images for different scanning and reconstruction protocols (X̅±s). **Table S3.** Comparison of SD values (HU) under different reconstruction algorithms for different tube voltage groups(X̅±s). **Table S4**. The correlation between radiation dose (CTDIvol) and background noise (SD values) in all reconstruction algorithms. **Table S5.** The correlation between the algorithm intensity of reconstruction and the low iodine concentration(1.25 mgI/mL) in terms of the CNR in all tube voltage groups. **Table S6.** Comparison of subjective image scores among different reconstruction algorithm groups (Friedman test). **Table S7.** Post hoc pairwise comparison of image quality scores for different reconstruction algorithm groups (120kV). **Table S8.** Post hoc pairwise comparison of image quality scores for different reconstruction algorithm groups (60kV).

## Data Availability

The datasets analyzed during the current study are available from the corresponding author on reasonable request.

## Abbreviations

CI: Clearinfinity
CNR: Contrast-to-noise ratio
CT: Computed tomography
CTDI_vol_: CT dose index volume
DLIR: Deep learning image reconstruction
FBP: Filtered back projection
IR: Iterative reconstruction
LoA: Limits of agreement
SD: Standard deviation

## References

[1] Frija G, Damilakis J, Paulo G, Loose R, Vano E (2021) Cumulative effective dose from recurrent CT examinations in Europe: proposal for clinical guidance based on an ESR EuroSafe Imaging survey. Eur Radiol 31:5514–5523. 10.1007/s00330-021-07696-1 33710370 10.1007/s00330-021-07696-1 PMC8270793

[2] Pozzessere C, von Garnier C, Beigelman-Aubry C (2023) Radiation exposure to low-dose computed tomography for lung cancer screening: should we be concerned. Tomography 9:166–177. 10.3390/tomography9010015 36828367 10.3390/tomography9010015 PMC9964027

[3] Lim Y, Kim JS, Lee HJ, Lee JK, Lee HA, Park C (2024) Image quality and lesion detectability of low-concentration iodine contrast and low radiation hepatic multiphase CT using a deep-learning-based contrast-boosting model in chronic liver disease patients. Diagnostics (Basel) 14:2308. 10.3390/diagnostics14202308 39451631 10.3390/diagnostics14202308 PMC11507254

[4] Hou P, Feng X, Chen Y et al (2025) Ultralow-dose hepatic computed tomography with a novel real-time deep learning-based noise reduction algorithm: a prospective cross-sectional analysis of image quality and lesion detection. Quant Imaging Med Surg 15:7006–7018. 10.21037/qims-2025-365 40785866 10.21037/qims-2025-365 PMC12332563

[5] Dabli D, Frandon J, Belaouni A et al (2022) Optimization of image quality and accuracy of low iodine concentration quantification as function of dose level and reconstruction algorithm for abdominal imaging using dual-source CT: a phantom study. Diagn Interv Imaging 103:31–40. 10.1016/j.diii.2021.08.004 34625394 10.1016/j.diii.2021.08.004

[6] Koetzier LR, Mastrodicasa D, Szczykutowicz TP et al (2023) Deep learning image reconstruction for CT: technical principles and clinical prospects. Radiology 306:e221257. 10.1148/radiol.221257 36719287 10.1148/radiol.221257 PMC9968777

[7] Yang C, Wang W, Cui D et al (2023) Deep learning image reconstruction algorithms in low-dose radiation abdominal computed tomography: assessment of image quality and lesion diagnostic confidence. Quant Imaging Med Surg 13:3161–3173. 10.21037/qims-22-1227 37179954 10.21037/qims-22-1227 PMC10167467

[8] Nagayama Y, Sakabe D, Goto M et al (2021) Deep learning-based reconstruction for lower-dose pediatric CT: technical principles, image characteristics, and clinical implementations. Radiographics 41:1936–1953. 10.1148/rg.2021210105 34597178 10.1148/rg.2021210105

[9] Franck C, Zhang G, Deak P, Zanca F (2021) Preserving image texture while reducing radiation dose with a deep learning image reconstruction algorithm in chest CT: a phantom study. Phys Med 81:86–93. 10.1016/j.ejmp.2020.12.005 33445125 10.1016/j.ejmp.2020.12.005

[10] Benz DC, Ersözlü S, Mojon FLA et al (2022) Radiation dose reduction with deep-learning image reconstruction for coronary computed tomography angiography. Eur Radiol 32:2620–2628. 10.1007/s00330-021-08367-x 34792635 10.1007/s00330-021-08367-x PMC8921160

[11] Jo GD, Ahn C, Hong J et al (2023) 75% radiation dose reduction using deep learning reconstruction on low-dose chest CT. BMC Med Imaging 23:129. 10.1186/s12880-023-01081-8 37697262 10.1186/s12880-023-01081-8 PMC10494344

[12] Quaia E, Kiyomi Lanza de Cristoforis E, Agostini E, Zanon C (2024) Computed tomography effective dose and image quality in deep learning image reconstruction in intensive care patients compared to iterative algorithms. Tomography 10:878–887. 10.3390/tomography10060069 10.3390/tomography10060069 PMC11209234 38921946

[13] Jung Y, Hur J, Han K et al (2023) Radiation dose reduction using deep learning-based image reconstruction for a low-dose chest computed tomography protocol: a phantom study. Quant Imaging Med Surg 13:2303–2315. 10.21037/qims-22-618 10.21037/qims-22-618 PMC10006148 36915339

[14] Noda Y, Kawai N, Kawamura T et al (2022) Radiation and iodine dose reduced thoraco-abdomino-pelvic dual-energy CT at 40 keV reconstructed with deep learning image reconstruction. Br J Radiol 95:20211163. 10.1259/bjr.20211163 35230135 10.1259/bjr.20211163 PMC10996425

[15] Greffier J, Dabli D, Hamard A et al (2021) Impact of dose reduction and the use of an advanced model-based iterative reconstruction algorithm on spectral performance of a dual-source CT system: a task-based image quality assessment. Diagn Interv Imaging 102:405–412. 10.1016/j.diii.2021.03.002 33820752 10.1016/j.diii.2021.03.002

[16] Choi M, Lee YJ, Jung SE (2022) The image quality and diagnostic performance of CT with low-concentration iodine contrast (240 mg iodine/mL) for the abdominal organs. Diagnostics (Basel) 12:752. 10.3390/diagnostics12030752 35328304 10.3390/diagnostics12030752 PMC8947528

[17] Lincoln JD, Parsons D, Clarke SE et al (2019) Technical note: evaluation of kV CBCT enhancement using a liver-specific contrast agent for stereotactic body radiation therapy image guidance. Med Phys 46:1175–1181. 10.1002/mp.13384 30624784 10.1002/mp.13384

[18] Yoshida K, Nagayama Y, Funama Y et al (2024) Low tube voltage and deep-learning reconstruction for reducing radiation and contrast medium doses in thin-slice abdominal CT: a prospective clinical trial. Eur Radiol 34:7386–7396. 10.1007/s00330-024-10793-6 38753193 10.1007/s00330-024-10793-6

[19] Li LL, Wang H, Song J, Shang J, Zhao XY, Liu B (2021) A feasibility study of realizing low-dose abdominal CT using deep learning image reconstruction algorithm. J Xray Sci Technol 29:361–372. 10.3233/XST-200826 33612538 10.3233/XST-200826

[20] Li W, Huang W, Li P et al (2024) Application of deep learning image reconstruction-high algorithm in one-stop coronary and carotid-cerebrovascular CT angiography with low radiation and contrast doses. Quant Imaging Med Surg 14:1860–1872. 10.21037/qims-23-864 38415146 10.21037/qims-23-864 PMC10895143

[21] Li W, Lu H, Wen Y et al (2023) Reducing both radiation and contrast doses for overweight patients in coronary CT angiography with 80-kVp and deep learning image reconstruction. Eur J Radiol 161:110736. 10.1016/j.ejrad.2023.110736 36804314 10.1016/j.ejrad.2023.110736

[22] Park HJ, Choi SY, Lee JE et al (2022) Deep learning image reconstruction algorithm for abdominal multidetector CT at different tube voltages: assessment of image quality and radiation dose in a phantom study. Eur Radiol 32:3974–3984. 10.1007/s00330-021-08459-8 35064803 10.1007/s00330-021-08459-8

[23] Lyu P, Li Z, Chen Y et al (2024) Deep learning reconstruction CT for liver metastases: low-dose dual-energy vs standard-dose single-energy. Eur Radiol 34:28–38. 10.1007/s00330-023-10033-3 37532899 10.1007/s00330-023-10033-3

[24] Masuda S, Yamada Y, Minamishima K, Owaki Y, Yamazaki A, Jinzaki M (2022) Impact of noise reduction on radiation dose reduction potential of virtual monochromatic spectral images: comparison of phantom images with conventional 120 kVp images using deep learning image reconstruction and hybrid iterative reconstruction. Eur J Radiol 149:110198. 10.1016/j.ejrad.2022.110198 35168172 10.1016/j.ejrad.2022.110198

[25] Frings M, Welsner M, Mousa C et al (2024) Low-dose high-resolution chest CT in adults with cystic fibrosis: intraindividual comparison between photon-counting and energy-integrating detector CT. Eur Radiol Exp 8:105. 10.1186/s41747-024-00502-9 39298080 10.1186/s41747-024-00502-9 PMC11413257

[26] Zhao Y, Luu N, Hubbard L, Malkasian S, Molloi S (2025) Pulmonary regional blood flow: validation of low-dose two-volume dynamic CT perfusion imaging in a swine model. Eur Radiol Exp 9:17. 10.1186/s41747-025-00556-3 39966217 10.1186/s41747-025-00556-3 PMC11836245

[27] van den Berk IAH, Jacobs C, Kanglie MMNP et al (2024) An AI deep learning algorithm for detecting pulmonary nodules on ultra-low-dose CT in an emergency setting: a reader study. Eur Radiol Exp 8:132. 10.1186/s41747-024-00518-1 39565453 10.1186/s41747-024-00518-1 PMC11579269

[28] Kawashima H, Ichikawa K, Matsubara K, Nagata H, Takata T, Kobayashi S (2019) Quality evaluation of image-based iterative reconstruction for CT: comparison with hybrid iterative reconstruction. J Appl Clin Med Phys 20:199–205. 10.1002/acm2.12597 31050148 10.1002/acm2.12597 PMC6560231

[29] van Stiphout JA, Driessen J, Koetzier LR et al (2022) The effect of deep learning reconstruction on abdominal CT densitometry and image quality: a systematic review and meta-analysis. Eur Radiol 32:2921–2929. 10.1007/s00330-021-08438-z 34913104 10.1007/s00330-021-08438-z PMC9038933

[30] Huang X, Zhao W, Wang G et al (2023) Improving image quality with deep learning image reconstruction in double-low-dose head CT angiography compared with standard dose and adaptive statistical iterative reconstruction. Br J Radiol 96:20220625. 10.1259/bjr.20220625 36606518 10.1259/bjr.20220625 PMC9975360

[31] Caruso D, De Santis D, Del Gaudio A et al (2024) Low-dose liver CT: image quality and diagnostic accuracy of deep learning image reconstruction algorithm. Eur Radiol 34:2384–2393. 10.1007/s00330-023-10171-8 37688618 10.1007/s00330-023-10171-8 PMC10957592

[32] Sato M, Ichikawa Y, Domae K et al (2022) Deep learning image reconstruction for improving image quality of contrast-enhanced dual-energy CT in abdomen. Eur Radiol 32:5499–5507. 10.1007/s00330-022-08647-0 35238970 10.1007/s00330-022-08647-0

[33] Kawai N, Noda Y, Nakamura F et al (2024) Low-tube-voltage whole-body CT angiography with extremely low iodine dose: a comparison between hybrid-iterative reconstruction and deep-learning image-reconstruction algorithms. Clin Radiol 79:e791–e798. 10.1016/j.crad.2024.02.002 38403540 10.1016/j.crad.2024.02.002

[34] Yamasaki Y, Kamitani T, Sagiyama K et al (2021) Model-based iterative reconstruction for 320-detector row CT angiography reduces radiation exposure in infants with complex congenital heart disease. Diagn Interv Radiol 27:42–49. 10.5152/dir.2020.19633 33290239 10.5152/dir.2020.19633 PMC7837718

[35] Bornet PA, Villani N, Gillet R et al (2022) Clinical acceptance of deep learning reconstruction for abdominal CT imaging: objective and subjective image quality and low-contrast detectability assessment. Eur Radiol 32:3161–3172. 10.1007/s00330-021-08410-x 34989850 10.1007/s00330-021-08410-x

[36] Solomon J, Lyu P, Marin D, Samei E (2020) Noise and spatial resolution properties of a commercially available deep learning-based CT reconstruction algorithm. Med Phys 47:3961–3971. 10.1002/mp.14319 32506661 10.1002/mp.14319

[37] Greffier J, Frandon J, Hamard A et al (2020) Impact of iterative reconstructions on image quality and detectability of focal liver lesions in low-energy monochromatic images. Phys Med 77:36–42. 10.1016/j.ejmp.2020.07.024 32771702 10.1016/j.ejmp.2020.07.024

[38] Šegota Ritoša D, Dodig D, Kovačić S et al (2025) The impact of weighting factors on dual-energy computed tomography image quality in non-contrast head examinations: phantom and patient study. Diagnostics (Basel) 15:180. 10.3390/diagnostics15020180 39857064 10.3390/diagnostics15020180 PMC11763815

[39] Huang M, Chen W, Zhu Y, Duan Q, Zhu Y, Zhang Y (2025) An adaptive weighted residual-guided algorithm for non-uniformity correction of high-resolution infrared line-scanning images. Sensors (Basel) 25:1511. 10.3390/s25051511 40096407 10.3390/s25051511 PMC11902343

[40] Zhou Z, Huber NR, Inoue A, McCollough CH, Yu L (2023) Multislice input for 2D and 3D residual convolutional neural network noise reduction in CT. J Med Imaging 10:014003. 10.1117/1.JMI.10.1.014003 10.1117/1.JMI.10.1.014003 PMC9888548 36743869

