# Supplementary material for: The impact of a novel deep learning reconstruction algorithm on image quality in ultralow-dose CT: a quantitative phantom study
Source: Eur Radiol Exp. 2026 Jun 8;10:86. doi: 10.1186/s41747-026-00751-w (PMC13247107; doi:10.1186/s41747-026-00751-w)
Supplement: Supplementary file 1 — Additional file 1: Table S1. 5-point Likert scale for subjective image quality assessment. Table S2. Subjective evaluation scores of images for different scanning and reconstruction protocols (X̅±s). Table S3. Comparison of SD values (HU) under different reconstruction algorithms for different tube voltage groups(X̅±s). Table S4. The correlation between radiation dose (CTDIvol) and background noise (SD values) in all reconstruction algorithms. Table S5. The correlation between the algorithm intensity of reconstruction and the low iodine concentration(1.25 mgI/mL) in terms of the CNR in all tube voltage groups. Table S6. Comparison of subjective image scores among different reconstruction algorithm groups (Friedman test). Table S7. Post hoc pairwise comparison of image quality scores for different reconstruction algorithm groups (120kV). Table S8. Post hoc pairwise comparison of image quality scores for different reconstruction algorithm groups (60kV). [file 41747_2026_751_MOESM1_ESM.pdf]

The impact of a novel deep learning reconstruction algorithm on image quality in ultralow-dose CT: a quantitative phantom study

ELECTRONIC SUPPLEMENTARY MATERIAL

Additional results data (Table S1-S8). Description: subjective evaluation criteria and results for image quality; statistical results comparing different reconstruction algorithms and different tube voltage groups

Table S1. 5-point Likert scale for subjective image quality assessment.

Score	Rating Level	Description
1	Unacceptable	Image quality is completely non-diagnostic.
2	Poor	Significant noise or artifacts substantially interfere with assessment.
3	Average	Image quality is adequate, with minor influence from noise or artifacts.
4	Good	Image quality is good, without obvious noise or artifacts.
5	Excellent	Image quality is outstanding, with no perceptible noise or artifacts.

Two radiologists independently evaluated the overall image quality based on the aforementioned defined standards, with the scoring criteria being the level of image noise and artifacts.

Table S2. Subjective evaluation scores of images for different scanning and reconstruction protocols ($\overline{X}$±s)

	FBP	CV50%	CI10%	CI30%	CI50%	CI70%	CI90%
**120 kV**	1.6±0.52	1.8±0.42	1.7±0.48	2.6±0.52	3.8±0.63*†	4.5±0.53*†	5.0±0.0*†
**100 kV**	1.3±0.48	1.5±0.53	1.6±0.52	2.4±0.52*	3.4±0.52*†	4.3±0.48*†	4.8±0.42*†
**80 kV**	1.0±0.00	1.4±0.52	1.4±0.52	2.3±0.48*	3.1±0.57*†	4.1±0.57*†	4.4±0.52*†
**60 kV**	1.0±0.00	1.1±0.32	1.1±0.32	1.7±0.48	2.2±0.42*†	3.3±0.48*†	3.6±0.52*†

* Compared with the FBP reconstruction algorithm under the same tube voltage conditions, $p < 0.05$;
† Compared with the CV50% reconstruction algorithm under the same tube voltage conditions, $p < 0.05$;
The rating range is 1-5 points, with higher scores indicating better image quality; The P-value has been corrected for multiple comparisons using the Bonferroni method; FBP, Filtered back projection; CV, ClearView; CI, ClearInfinity;

Table S3. Comparison of SD values (HU) under different reconstruction algorithms for different tube voltage groups($\bar{X}$±s)

	FBP	CV50%	CI10%	CI30%	CI50%	CI70%	CI90%	*P*
Background SD (0 mgI/mL)								
120kV	12.94±0.95	9.08±0.71	11.50±0.95	8.50±0.66	5.91±0.44	3.94±0.30	2.81±0.36	<0.001
100kV	14.81±1.02	9.98±0.66	12.21±0.62	9.21±0.55	6.57±0.36	3.97±0.29	3.02±0.37	<0.001
80kV	16.45±1.05	11.61±0.91	13.78±1.10	10.67±0.74	7.53±0.53	4.46±0.39	3.57±0.51	<0.001
60kV	23.74±1.57	13.89±1.29	15.87±1.15	11.71±1.22	8.34±0.81	4.74±0.54	3.63±0.48	<0.001
SD (1.25 mgI/mL)								
120kV	12.61±0.78	8.92±0.48	11.1±0.45	8.29±0.51	5.90±0.36	3.46±0.24	2.64±0.24	<0.001
100kV	15.04±0.69	10.26±0.44	12.27±0.65	9.56±0.54	6.70±0.27	3.87±0.24	2.83±0.22	<0.001
80kV	17.37±2.15	12.27±1.38	14.46±1.53	11.3±1.15	8.00±0.94	4.81±0.52	3.41±0.27	<0.001
60kV	23.09±2.94	13.68±1.40	15.2±1.71	11.65±1.32	8.67±1.14	4.91±0.63	4.19±0.76	<0.001
SD (5 mgI/mL)								
120kV	12.35±0.73	8.76±0.49	11.10±0.45	8.24±0.49	5.90±0.36	3.46±0.24	2.61±0.23	<0.001
100kV	16.38±0.97	11.32±0.94	12.26±0.65	10.13±0.64	6.69±0.27	3.87±0.24	2.81±0.22	<0.001
80kV	16.51±1.43	11.86±1.22	14.46±1.53	10.88±0.91	7.99±0.94	4.80±0.52	3.41±0.27	<0.001
60kV	27.28±3.41	16.75±2.53	15.20±1.71	14.11±1.61	8.66±1.14	4.91±0.63	4.19±0.76	<0.001
SD (10 mgI/mL)								
120kV	15.50±8.40	9.60±0.94	10.98±3.18	9.06±0.71	6.53±0.48	4.17±0.38	16.39±0.97	<0.001
100kV	16.13±1.42	11.22±1.03	13.62±1.30	10.30±0.62	7.75±0.82	5.02±0.62	3.75±0.39	<0.001
80kV	17.57±1.46	12.62±1.15	14.58±1.26	11.82±0.97	8.99±0.99	6.27±0.82	4.77±0.42	<0.001
60kV	28.14±3.39	15.58±2.04	17.08±2.20	14.15±1.47	11.31±1.37	8.95±1.29	6.80±0.83	<0.001

Table S4. The correlation between radiation dose (CTDIvol) and background noise (SD values) in all reconstruction algorithms

Reconstruction Algorithm	CTDIvol vs. Background noise(SD)	
	Spearman's ρ	*P* Value
FBP	-0.928	<0.001
CV50%	-0.908	<0.001
CI10%	-0.878	<0.001
CI30%	-0.851	<0.001
CI50%	-0.885	<0.001
CI70%	-0.789	<0.001
CI90%	-0.629	<0.001

Table S5. The correlation between the algorithm intensity of reconstruction and the low iodine concentration(1.25 mgI/mL) in terms of the CNR in all tube voltage groups

Tube Voltage	Reconstruction Algorithm vs. CNR (1.25 mgI/mL)	
	Spearman's ρ	*P* Value
120kV	0.924	<0.001
100kV	0.937	<0.001
80kV	0.930	<0.001
60kV	0.937	<0.001

Table S6. Comparison of subjective image scores among different reconstruction algorithm groups (Friedman test)

Tube Voltage	χ^2	*P* Value
120 kV	257.95	<0.001
100 kV	255.24	<0.001
80 kV	258.42	<0.001
60 kV	252.39	<0.001

Table S7. Post hoc pairwise comparison of image quality scores for different reconstruction algorithm groups (120kV)

Reconstruction Algorithm	Z	Original *p* value	Corrected *p* value*
FBP vs CI50%	-3.905	<0.001	<0.001
FBP vs CI70%	-3.907	<0.001	<0.001
FBP vs CI90%	-4.119	<0.001	<0.001
CV50% vs CI50%	-3.969	<0.001	<0.001
CV50% vs CI70%	-3.971	<0.001	<0.001
CV50% vs CI90%	-4.194	<0.001	<0.001

Using the Bonferroni correction, the significance level is set as α' = 0.05/21 ≈ 0.00238

Table S8. Post hoc pairwise comparison of image quality scores for different reconstruction algorithm groups (60kV)

**Reconstruction Algorithm**	**Z**	**Original *p* value**	**Corrected *p* value***
FBP vs CI50%	-4.194	<0.001	<0.001
FBP vs CI70%	-4.147	<0.001	<0.001
FBP vs CI90%	-4.119	<0.001	<0.001
CV50% vs CI50%	-3.843	<0.001	<0.001
CV50% vs CI70%	-4.065	<0.001	<0.001
CV50% vs CI90%	-4.038	<0.001	<0.001

Using the Bonferroni correction, the significance level is set as α’ = 0.05/21 ≈ 0.00238
